# Supplementary material for: The tyrosine kinase KDR is essential for the survival of HTLV-1-infected T cells by stabilizing the Tax oncoprotein
Source: Nat Commun. 2024 Jun 25;15:5380. doi: 10.1038/s41467-024-49737-5 (PMC11199648; doi:10.1038/s41467-024-49737-5)
Supplement: Supplementary file 1 — Supplementary Information [file 41467_2024_49737_MOESM1_ESM.pdf]

Supplementary Information

The tyrosine kinase KDR is essential for the survival of HTLV-1-infected T cells by stabilizing the Tax oncoprotein

Suchitra Mohanty[1], Sujit Suklabaidya[1], Alfonso Lavorgna[2, *], Takaharu Ueno[3], Jun-ichi Fujisawa[3], Nyater Ngouth[4], Steven Jacobson[4] and Edward W. Harhaj[1,#]

[1]Department of Microbiology and Immunology, Penn State College School of Medicine, Hershey, PA 17033, USA

[2]Department of Oncology, Sidney Kimmel Comprehensive Cancer Center, Johns Hopkins School of Medicine, Baltimore, MD 21287, USA

[3]Department of Microbiology, Kansai Medical University, Osaka, Japan 573-1010

[4]Viral Immunology Section, National Institute of Neurological Disorders and Stroke, National Institutes of Health, Bethesda, MD 20892, USA

[*]Current address: Millipore-Sigma, Rockville, MD 20850

[#]To whom correspondence should be addressed: ewh110@psu.edu

This Supplementary Information file contains:

Supplementary Figures 1-14

Supplementary Table 1

Supplementary Figure 1

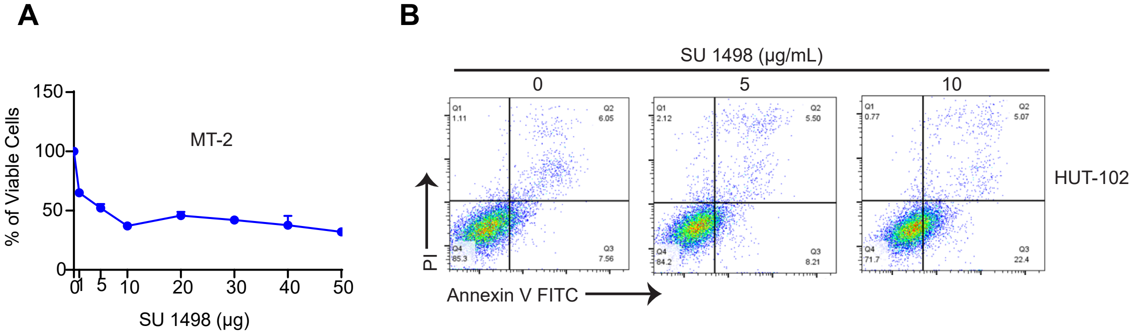

Supplementary Figure 1. KDR inhibition induces apoptosis in HTLV-1-transformed cells. **(A)** Cell viability assay was performed using MT-2 cells treated with different doses of SU 1498 for 24 hours. **(B)** Annexin V flow cytometry-based apoptosis assay was performed using HUT-102 cells treated with the indicated concentrations of SU 1498 for 24 hours.

Supplementary Figure 2

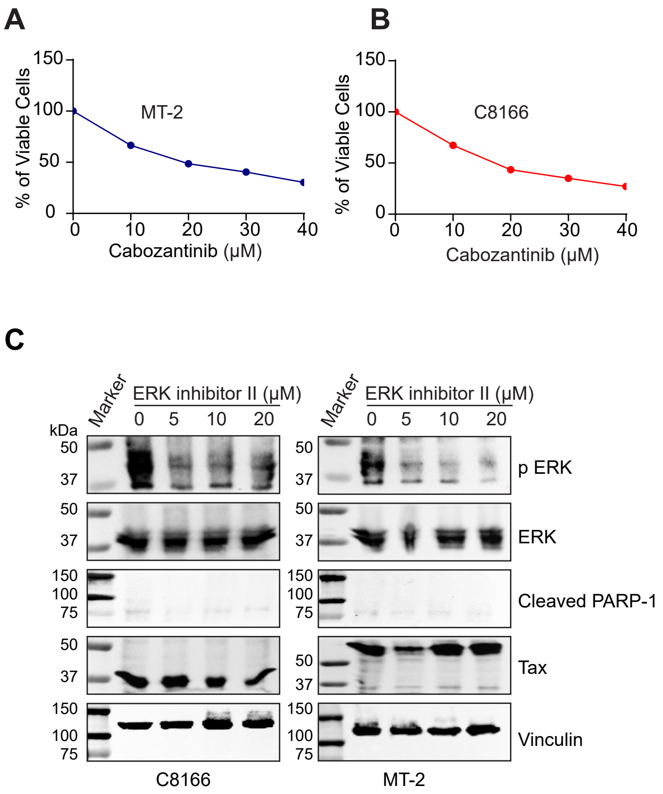

Supplementary Figure 2. Specificity of KDR inhibition in Tax degradation. (A and B) Cell viability assay was performed using MT-2 and C8166 cells treated with different doses of Cabozantinib. **(C)** Immunoblotting was performed with the indicated antibodies using lysates of C8166 and MT-2 cells treated with the ERK inhibitor II for 24 hours.

Supplementary Figure 3

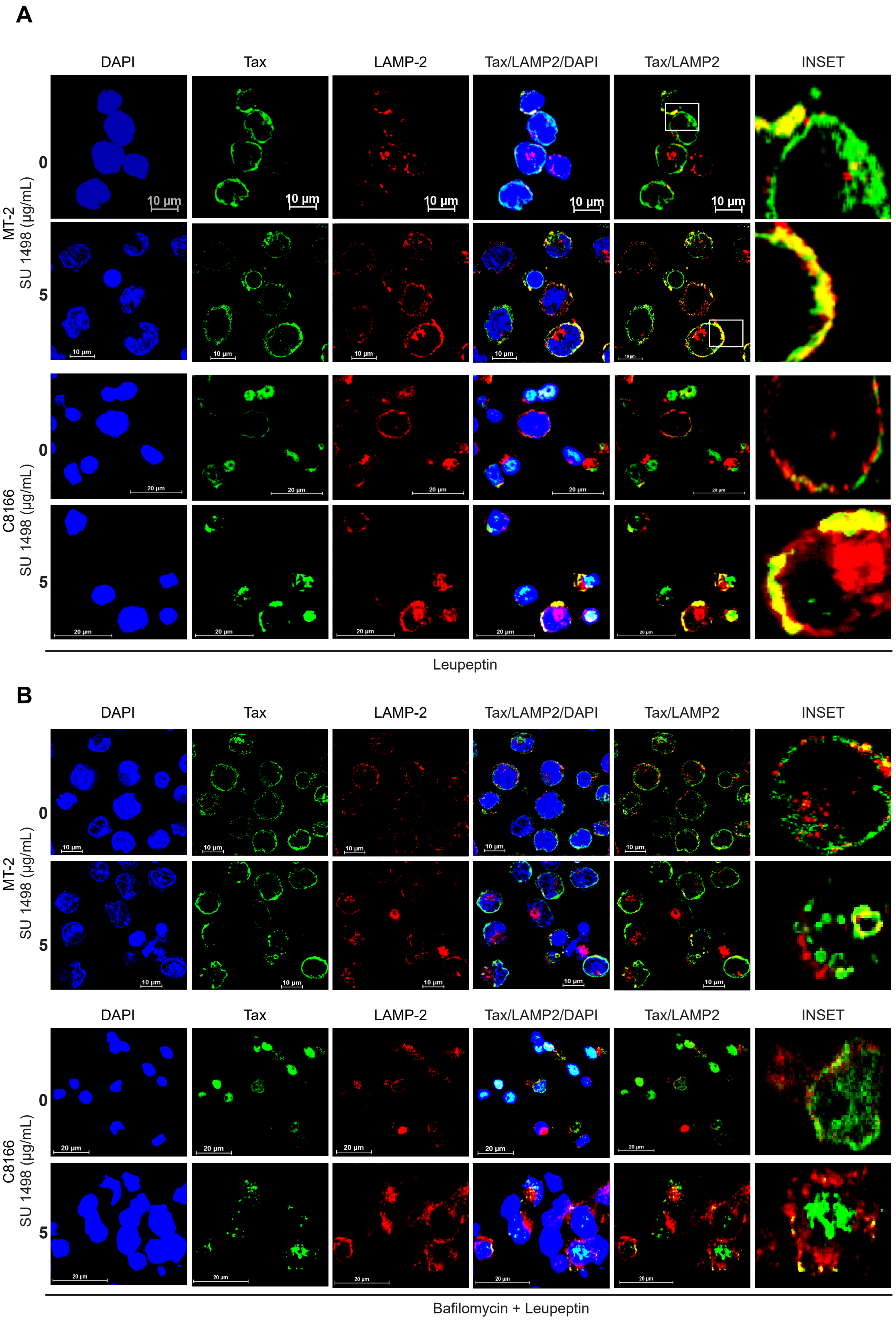

Supplementary Figure 3. Autophagic/lysosomal degradation of Tax (A) Immunofluorescence confocal microscopy was performed using MT-2 and C8166 cells treated with SU 1498 and leupeptin (20 nM) for 24 hours and labelled with Tax-Alexa Fluor 488 and LAMP2-Alexa Fluor 647 antibodies and DAPI for nuclear staining. **(B)** Immunofluorescence confocal microscopy was performed using MT-2 and C8166 cells treated with SU 1498, leupeptin (20 nM) and Bafilomycin A1 (20 nM) for 24 hours and labelled with Tax-Alexa Fluor 488 and LAMP2-Alexa Fluor 647 antibodies and DAPI for nuclear staining.

Supplementary Figure 4

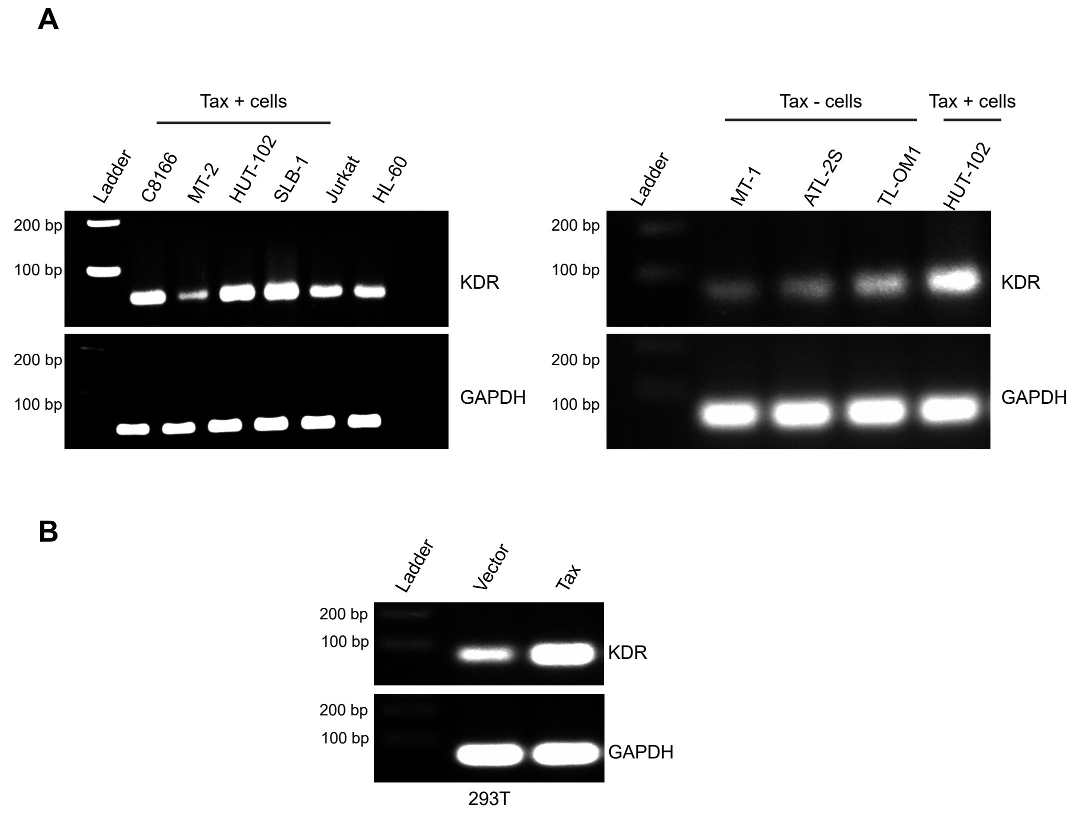

Supplementary Figure 4. KDR mRNA expression in Tax+ HTLV-1-transformed, Tax- ATLL, and Tax transfected 293T cells. (A) qRT-PCR of KDR mRNA in C8166, MT-2, HUT-102, SLB-1, Jurkat, HL-60, MT-1, ATL-2S, and TL-OM1 cell lines. **(B)** qRT-PCR of KDR mRNA in 293T cells transiently transfected with Tax.

Supplementary Figure 5

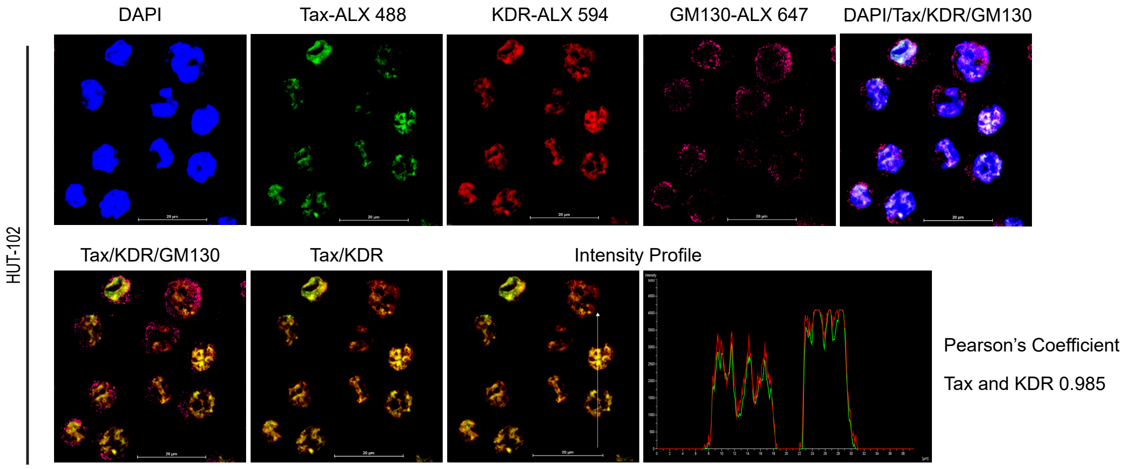

Supplementary Figure 5. Tax-KDR colocalization in HUT-102 cells. Immunofluorescence confocal microscopy was performed using HUT-102 cells with the indicated antibodies. The fluorescence intensity profile is plotted along the white arrow represented in the graph showing overlap, and Pearson's coefficient analysis was performed using NIS Element software.

Supplementary Figure 6

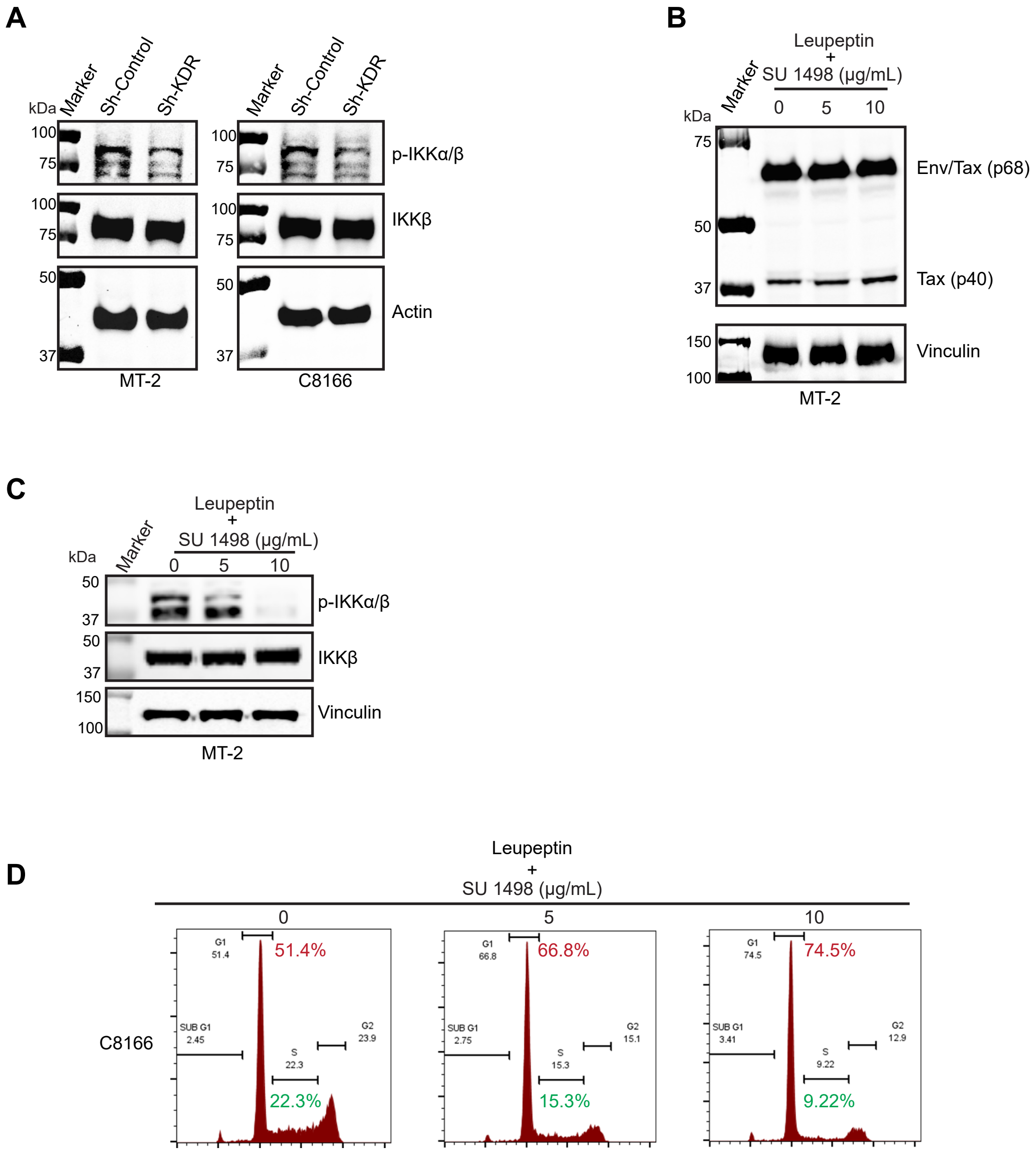

Supplementary Figure 6. KDR inhibition impairs NF-κB signaling and cell proliferation (A) Immunoblotting was performed with the indicated antibodies using lysates from MT-2 and C8166 cells expressing control scrambled shRNA or KDR shRNA. **(B-C)** Immunoblotting was performed with the indicated antibodies using lysates from MT-2 cells treated with SU 1498 and leupeptin (20 nM) for 24 hours. **(D)** Cell cycle analysis of C8166 cells treated with the indicated concentrations of SU1498 and leupeptin for 24 hours and the proportions of cells at different phases of the cell cycle (sub G1, G1/M, S, G2/M) were gated for analysis.

Supplementary Figure 7

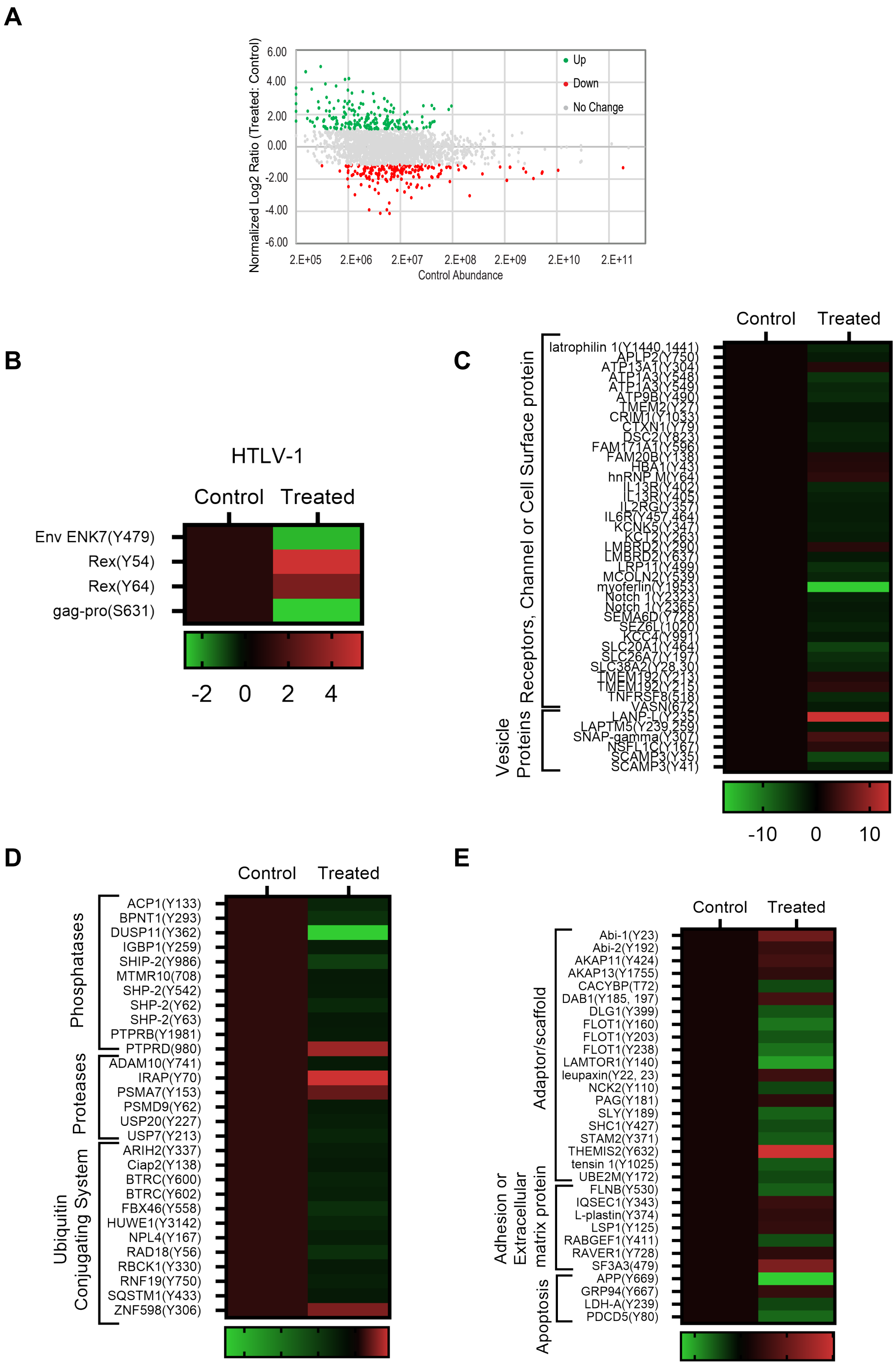

Supplementary Figure 7. KDR inhibition alters the tyrosine phosphorylation of viral and cellular proteins in MT-2 cells. (A) Phosphoproteomics results showing the fold change in total analyzed proteins in SU 1498-treated MT-2 cells. **(B-E)** Heatmap representation of phosphoproteomics results in SU 1498-treated MT-2 cells showing alterations in tyrosine phosphorylation of viral and cellular proteins grouped by function. The color gradient indicates the intensity of gene expression.

Supplementary Figure 8

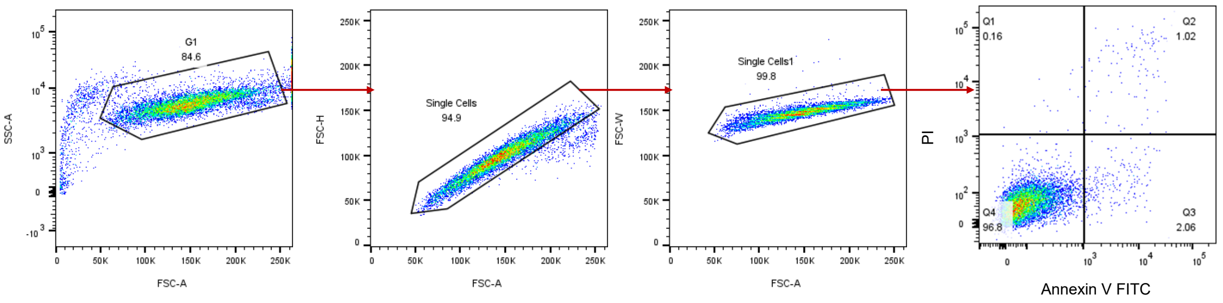

Supplementary Figure 8. Gating strategy for the Annexin V FITC assay. A gate was set on the FSC and SSC parameters based on their size and granularity to select cells of interest and eliminate debris. Gating was then performed on FSC-H and FSC-A dot plots, and further gating was set on FSC-H and FSC-W dot plots to eliminate doublets. The final quadrant gate was set using a negative control to evaluate the induction of apoptosis. The identical gating strategy was applied to all the samples.

Supplementary Figure 9

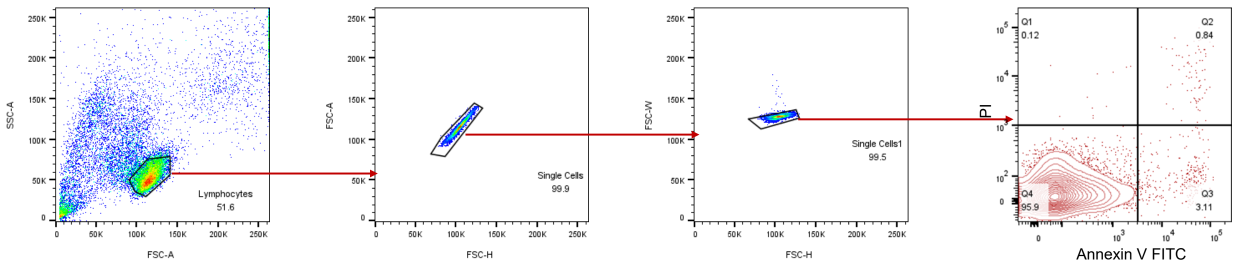

Supplementary Figure 9. Gating strategy for Annexin V FITC staining in PBMCs. A gate was set on the FSC and SSC parameters based on the size and granularity of the lymphocytes. Next, gating was performed on FSC-H and FSC-A dot plots, and further gating was set on FSC-H and FSC-W dot plots to eliminate doublets. The final quadrant gate was set by using a negative control to confirm apoptosis induction. The identical gating strategy was applied to all the samples.

Supplementary Figure 10

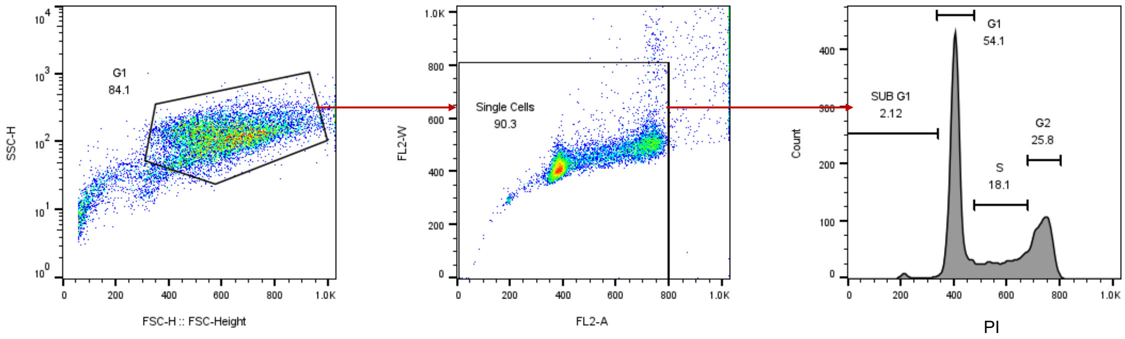

Supplementary Figure 10. Gating strategy for cell cycle analysis. A gate was set on the FSC and SSC parameters based on their size and granularity to select cells of interest by eliminating debris. Further gating was set on FL2-A and FL2-W to eliminate doublets, and the final gates were applied to assign the sub G1, G1, S, and G2/M phases of the cell cycle based on the DNA content/PI staining of an untreated sample. The identical gating strategy was applied to all the samples.

Supplementary Figure 11

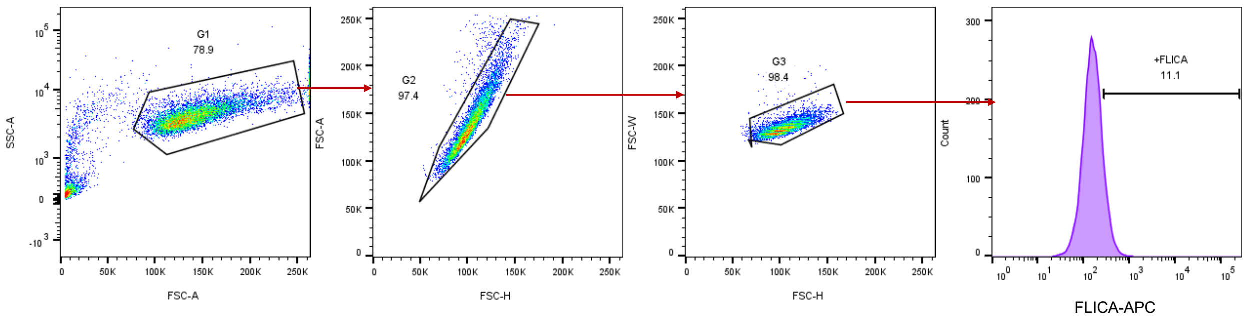

Supplementary Figure 11. Gating strategy for the FLICA caspase assay. A gate was set on the FSC and SSC parameters to select the cells of interest and eliminate debris based on their size and granularity. Gating was then performed on FSC-H and FSC-A dot plots and further gating was set on FSC-H and FSC-W dot plots to eliminate doublets. The final gate was set on FLICA-APC-positive cells using a negative control (unstained) to examine caspase activation. The identical gating strategy was applied to all the samples.

Supplementary Figure 12

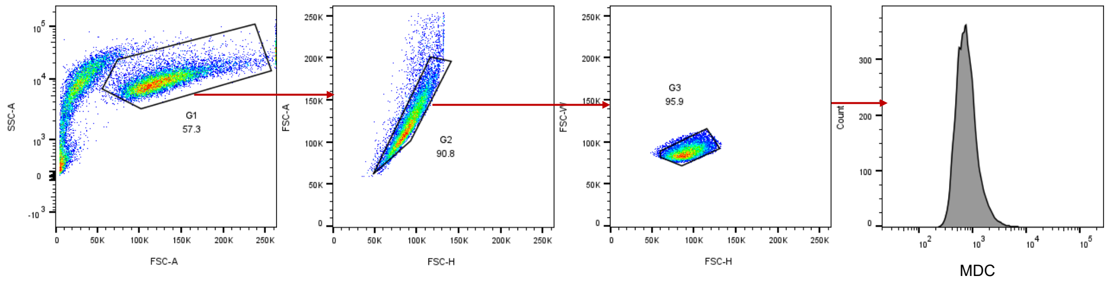

Supplementary Figure 12. Gating strategy for autophagolysosome detection by MDC staining. A gate was set on FSC and SSC parameters based on size and granularity to select cells of interest and eliminate cell debris. Gating was then performed on FSC-H and FSC-A dot plots, and further gating was set on FSC-H and FSC-W dot plots to eliminate doublets. A histogram was displayed with MDC-positive cells using a negative (unstained) control to examine autophagy induction. The identical gating strategy was applied to all the samples.

Supplementary Figure 13

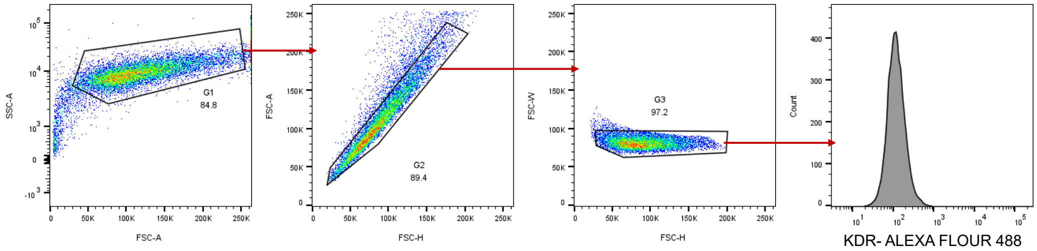

Supplementary Figure 13. Gating strategy for KDR-Alexa Fluor 488 staining. A gate was set on the FSC and SSC parameters based on size and granularity to select cells of interest by eliminating debris. Gating was then performed on FSC-H and FSC-A dot plots and further gating was set on FSC-H and FSC-W dot plots to eliminate doublets. A histogram was displayed with Alexa Fluor 488 positive cells using a negative control to examine KDR expression. The identical gating strategy was applied to all the samples.

Supplementary Figure 14

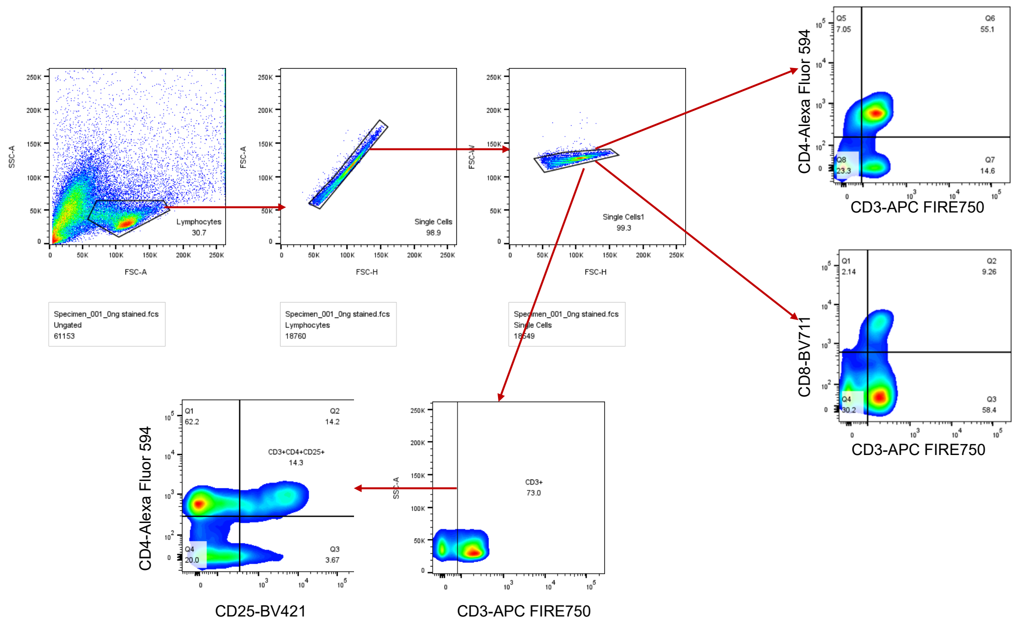

Supplementary Figure 14. Gating strategy for CD3+, CD4+, CD25+ and CD8+ cells in PBMCs. A gate was set on FSC and SSC parameters based on size and granularity to select lymphocytes. Next, gating was performed on FSC-H and FSC-A dot plots and further gating was set on FSC-H and FSC-W dot plots to eliminate doublets. The quadrant gate was set on CD3+CD4+ and CD3+CD8+ positive cells using a negative (isotype) control. Only CD3+ cells were gated using the negative (isotype) control, and the final gate was set on CD3+ cells with CD3+CD4+CD25+ cells using the negative (isotype) control. The identical gating strategy was applied to all the samples.

Supplementary Table 1

Patient #	Gender	Age (years)	HTLV-1 PVL (%) in PBMCs
HAM #1	Male	66	42.5
HAM #2	Male	66	21.5
HAM #3	Female	57	10.3
Control #1	Female	32	N/A
Control #2	Female	28	N/A
Control #3	Female	24	N/A

Supplementary Table 1. Demographics of healthy controls and HAM/TSP patients. The gender, age and HTLV-1 proviral loads (PVLs) are indicated.
