# Supplementary material for: The tyrosine kinase KDR is essential for the survival of HTLV-1-infected T cells by stabilizing the Tax oncoprotein
Source: Nat Commun. 2024 Jun 25;15:5380. doi: 10.1038/s41467-024-49737-5 (PMC11199648; doi:10.1038/s41467-024-49737-5)
Supplement: Supplementary file 3 — Description of Additional Supplementary Files [file 41467_2024_49737_MOESM3_ESM.pdf]

Description of Additional Supplementary Files

File Name: Supplementary Data 1
Description: Related to Figure 1A-C. Kinome-wide shRNA screen in MT-2 cells. Values from CellTiter Glo assay.

File Name: Supplementary Data 2
Description: Related to Figure 1A-C. Kinome-wide shRNA screen in MT-2 cells. Values from CellTiter Glo assay from individual 96-well plates.

File Name: Supplementary Data 3
Description: Related to Figure 7C. Details of tyrosine phosphoproteomics data from MT-2 cells treated with SU 1498.
