# Supplementary material for: The tyrosine kinase KDR is essential for the survival of HTLV-1-infected T cells by stabilizing the Tax oncoprotein
Source: Nat Commun. 2024 Jun 25;15:5380. doi: 10.1038/s41467-024-49737-5 (PMC11199648; doi:10.1038/s41467-024-49737-5)
Supplement: Supplementary file 7 — Reporting Summary [file 41467_2024_49737_MOESM7_ESM.pdf]

nature portfolio

Corresponding author(s): Edward W Harhaj, Ph.D.

Last updated by author(s): 05/08/2024

Reporting Summary

Nature Portfolio wishes to improve the reproducibility of the work that we publish. This form provides structure for consistency and transparency in reporting. For further information on Nature Portfolio policies, see our Editorial Policies and the Editorial Policy Checklist.

Statistics

For all statistical analyses, confirm that the following items are present in the figure legend, table legend, main text, or Methods section.

n/a	Confirmed	
☐	☒	The exact sample size (*n*) for each experimental group/condition, given as a discrete number and unit of measurement
☐	☒	A statement on whether measurements were taken from distinct samples or whether the same sample was measured repeatedly
☐	☒	The statistical test(s) used AND whether they are one- or two-sided<br>*Only common tests should be described solely by name; describe more complex techniques in the Methods section.*
☒	☐	A description of all covariates tested
☐	☒	A description of any assumptions or corrections, such as tests of normality and adjustment for multiple comparisons
☐	☒	A full description of the statistical parameters including central tendency (e.g. means) or other basic estimates (e.g. regression coefficient) AND variation (e.g. standard deviation) or associated estimates of uncertainty (e.g. confidence intervals)
☐	☒	For null hypothesis testing, the test statistic (e.g. *F*, *t*, *r*) with confidence intervals, effect sizes, degrees of freedom and *P* value noted<br>*Give P values as exact values whenever suitable.*
☒	☐	For Bayesian analysis, information on the choice of priors and Markov chain Monte Carlo settings
☒	☐	For hierarchical and complex designs, identification of the appropriate level for tests and full reporting of outcomes
☐	☒	Estimates of effect sizes (e.g. Cohen's *d*, Pearson's *r*), indicating how they were calculated

Our web collection on statistics for biologists contains articles on many of the points above.

Software and code

Policy information about availability of computer code

Data collection	qRT-PCR data were collected using a QuantStudio 3 (Applied Biosystems).<br>Western blot images were acquired using an Azure 600 and Bio-Rad ChemiDoc.<br>Flow cytometry data was acquired using a BD FACS Symphony A3 and FACS Calibur (BD Biosciences).<br>Confocal images were acquired using a C2+ confocal microscope (Nikon).<br>Live cell imaging was performed with an Incucyte S3 imaging system (Sartorius).<br>LC-MS/MS analysis was performed with an OrbiTrap-Fusion Lumos Tribid (Thermo Scientific).
Data analysis	Flow Cytometry data was acquired using BDFACS Diva and BD CellQuest software and analyzed using FlowJo v10.<br>Western blot images were processed using Image Lab software 6.1 (Bio-Rad Laboratories).<br>Statistical analysis was performed using GraphPad Prism 10.<br>Mander's or Pearson's correlation was calculated using NIS-Elements AR 4.30.01 analysis software.<br>Confocal images were acquired using NIS-Elements AR 5.21.03 and images were processed using NIS-Elements AR 4.30.01 analysis software.<br>MS/MS spectra were evaluated with GFY-Core, version 3.8.<br>Acquisition and analysis of Incucyte S3 images was performed with Incucyte S3 software v2021C.<br>Scale bars were added to Incucyte images using (Fiji is just) Image J 2.14. 0/1.54F.<br>Figures were prepared with Adobe Illustrator version 28.1.

For manuscripts utilizing custom algorithms or software that are central to the research but not yet described in published literature, software must be made available to editors and reviewers. We strongly encourage code deposition in a community repository (e.g. GitHub). See the Nature Portfolio guidelines for submitting code & software for further information.

Data

Policy information about availability of data

All manuscripts must include a data availability statement. This statement should provide the following information, where applicable:

- Accession codes, unique identifiers, or web links for publicly available datasets
- A description of any restrictions on data availability
- For clinical datasets or third party data, please ensure that the statement adheres to our policy

All data generated to support the findings of this study are available within the paper, either in main or supplemental figures. The raw mass spec data has been deposited to the ProteomeXchange Consortium via the PRIDE partner repository with the accession number PXD051981.

Research involving human participants, their data, or biological material

Policy information about studies with human participants or human data. See also policy information about sex, gender (identity/presentation), and sexual orientation and race, ethnicity and racism.

Reporting on sex and gender	PBMCs were obtained from three healthy controls and three HAM/TSP patients. The healthy controls were all females. HAM/TSP patients consisted of two males and one female. Sex/gender was not considered in the study design due to the small number of patients.
Reporting on race, ethnicity, or other socially relevant groupings	No information about race or ethnicity of the patients was provided.
Population characteristics	HAM/TSP patients:<br>1) Male 66y/o Proviral load (PVL) % in PBMCs: 42.50%<br>2) Male 66 y/o PVL 21.50%<br>3) Female 57 y/o PVL 10.3%<br><br>Healthy controls:<br>1) Female 32 y/o<br>2) Female 28 y/o<br>3) Female 24 y/o
Recruitment	N/A
Ethics oversight	Blood samples from HAM/TSP patients were collected under protocol# 98N0047 approved by the National Institutes of Health IRB #10 (the NIH Intramural IRB), IRB Registration: IRB00011862 and the National Institute of Neurologic Disorders and Stroke (NINDS) Scientific Review Committee. Prior to study inclusion, written informed consent was obtained from subjects in accordance with the Declaration of Helsinki.

Note that full information on the approval of the study protocol must also be provided in the manuscript.

Field-specific reporting

Please select the one below that is the best fit for your research. If you are not sure, read the appropriate sections before making your selection.

[x] Life sciences   [ ] Behavioural & social sciences   [ ] Ecological, evolutionary & environmental sciences

For a reference copy of the document with all sections, see nature.com/documents/nr-reporting-summary-flat.pdf

Life sciences study design

All studies must disclose on these points even when the disclosure is negative.

Sample size	Sample sizes were not predetermined. The sample sizes was determined based on our previous publications and other relevant studies (PMID: 33362245; PMID: 25340740; PMID: 35219733). For experiments with clinical samples, a total of three healthy controls and three HAM/TSP patients were available for our studies.
Data exclusions	No data was excluded from analysis.
Replication	All results are reproducible and experiments were performed a minimum of two times.
Randomization	For immunofluorescence experiments, images were acquired from random areas in a given field.
Blinding	Blinding was not relevant to our study due to the types of experiments (i.e., western blotting, flow cytometry) performed in the manuscript .

Reporting for specific materials, systems and methods

We require information from authors about some types of materials, experimental systems and methods used in many studies. Here, indicate whether each material, system or method listed is relevant to your study. If you are not sure if a list item applies to your research, read the appropriate section before selecting a response.

Materials & experimental systems

n/a	Involved in the study
☐	☒ Antibodies
☐	☒ Eukaryotic cell lines
☒	☐ Palaeontology and archaeology
☒	☐ Animals and other organisms
☒	☐ Clinical data
☒	☐ Dual use research of concern
☒	☐ Plants

Methods

n/a	Involved in the study
☒	☐ ChIP-seq
☐	☒ Flow cytometry
☒	☐ MRI-based neuroimaging

Antibodies

Antibodies used

Alpha Tubulin Ab (Santa Cruz Biotechnology Cat#sc-5286) Dilution 1:1000
Vinculin Ab (Santa Cruz Biotechnology Cat#sc-73614) Dilution 1:3000
Beta Actin Ab (Santa Cruz Biotechnology Cat#sc-47778) Dilution 1:6000
Cleaved PARP (Asp214) rabbit mAb (Cell Signaling Cat#5625) Dilution 1:1000
Flk-1/KDR/VEGFR2 Ab (Santa Cruz Biotechnology Cat#sc-6251) Dilution 1:1000
VEGFR2 rabbit mAb (Cell Signaling Cat#2479) Dilution 1:1000 for western blot, Dilution 1:500 for Immunofluorescence and 1:100 for flowcytometry
NEMO/IKKgamma Ab (Santa Cruz Biotechnology Cat#sc-8032) Dilution 1:1000
IKKb rabbit mAb (Cell Signaling Cat#8943) Dilution 1:2000
pIKKa/b (Ser176/180) rabbit mAb (Cell Signaling Cat#2697) Dilution 1:1000
pIkBa mouse mAb (Cell Signaling Cat#9246) Dilution 1:1000
IkBa rabbit mAb (Cell Signaling Cat#4812) Dilution 1:1000
HTLV-1 Tax Ab (Santa Cruz Biotechnology Cat#sc-57872)Dilution 1:1000 for western blot and Dilution 1:500 for Immunofluorescence
HTLV-1 p19 Ab (ZeptoMetrix Cat#801003) Dilution 1:1000
pTyrosine mouse mAb (Cell Signaling Cat#9411) Dilution 1:1000
pJAK1 (Tyr1034/1035) rabbit mAb (Cell Signaling Cat#74129) Dilution 1:1000
pJAK2 (Tyr1008) rabbit mAb (Cell Signaling Cat#8082)Dilution 1:1000
pJAK3 (Tyr980/981) rabbit mAb (Cell Signaling Cat#5031)Dilution 1:1000
JAK1 rabbit mAB (Cell Signaling Cat#3344)Dilution 1:1000
JAK2 rabbit mAb (Cell Signaling Cat#3230)Dilution 1:1000
JAK3 rabbit mAb (Cell Signaling Cat#8827)Dilution 1:1000
pSTAT1 (Tyr701) rabbit mAb (Cell Signaling Cat#7649)Dilution 1:1000
pSTAT3 (Tyr705) rabbit mAb (Cell Signaling Cat#9145)Dilution 1:1000
pSTAT3 (Ser727) Ab (Cell Signaling Cat#9134)Dilution 1:1000
STAT1 rabbit mAb (Cell Signaling Cat#14994)Dilution 1:1000
STAT3 rabbit mAb (Cell Signaling Cat#4904)Dilution 1:1000
pVEGFR2 (Tyr1175) (Cell Signaling Cat#2478)Dilution 1:500 for Immunofluorescence
pERK (Thr202/Tyr204) rabbit mAb (Cell Signaling Cat#4370) Dilution 1:1000
ERK rabbit mAb (Cell Signaling Cat#4695) Dilution 1:1000
Rabbit IgG HRP Linked Whole Ab (Cytiva Cat# NA934-1ML) Dilution 1:5000
Mouse IgG HRP Linked Whole Ab (Cytiva Cat# NA931-1ML) Dilution 1:5000
GM130 polyclonal antibody, Alexa Fluor 647 (Thermo Fisher Scientific Cat#PA1-077-A647) Dilution 1:300
LAMP2 monoclonal antibody, Alexa Fluor 647 (Thermo Fisher Scientific Cat#A15464) Dilution 1:100
Goat anti-Rabbit (H+L) Cross-Adsorbed Secondary Antibody, Alexa Fluor 488 (Thermo Fisher Scientific Cat#A-11008) Dilution 1:1000
Goat anti-Mouse (H+L) Cross-Adsorbed Secondary Antibody, Alexa Fluor 594 (Thermo Fisher Scientific Cat#A-11005) Dilution 1:1000
Alexa Fluor 594 anti-human CD4 (BioLegend Cat#300544) Dilution 1:50
Alexa Fluor® 594 Mouse IgG1, κ Isotype Ctrl (Biolegend Cat#400174) Dilution 1:50
APC/Fire 750 anti-human CD3 (BioLegend Cat#317352) Dilution 1:20
APC/Fire 750 Mouse IgG2a, k isotype control (BioLegend Cat#400284) Dilution 1:20
Brilliant Violet 421 anti-human CD25 (BioLegend Cat#302630) Dilution 1:20
Brilliant Violet 421™ Mouse IgG1, κ Isotype (BioLegend Cat# 400158)Dilution 1:20
Brilliant Violet 711 anti-human CD8a (BioLegend Cat#301044) Dilution 1:20
Brilliant Violet 711™ Mouse IgG1,κ Isotype Ctrl (BioLegend Cat#400168) Dilution 1:20
Annexin V Alexa Fluor 488 ready flow conjugate (Thermo Fisher Scientific Cat#R37174) 1 drop/sample

Validation

Antibodies used were individually validated by the manufacturer or in other studies.

Cell Signaling antibodies are validated by six complementary strategies as described on their website: https://www.cellsignal.com/about-us/cst-antibody-validation-principles
For all other antibodies refer to the manufacturer's website:
Alpha Tubulin Ab (Santa Cruz Biotechnology Cat#sc-5286); Manufacturer's website: https://www.scbt.com/p/alpha-tubulin-antibody-b-7
Vinculin Ab (Santa Cruz Biotechnology Cat#sc-73614); Manufacturer's website: https://www.scbt.com/p/vinculin-antibody-7f9
Beta Actin Ab (Santa Cruz Biotechnology Cat#sc-47778); Manufacturer's website: https://www.scbt.com/p/beta-actin-antibody-c4
Flk-1/KDR/VEGFR2 Ab (Santa Cruz Biotechnology Cat#sc-6251); Manufacturer's website: https://www.scbt.com/p/vegfr2-antibody-a-3
NEMO/IKKgamma Ab (Santa Cruz Biotechnology Cat#sc-8032); Manufacturer's website: https://www.scbt.com/p/ikkgamma-antibody-b-3
HTLV-1 Tax Ab (Santa Cruz Biotechnology Cat#sc-57872); Manufacturer's website: https://www.scbt.com/p/htlv-1-tax-antibody-1a3
HTLV-1 p19 Ab (ZeptoMetrix Cat#801003); Manufacturer's website: https://www.zeptometrix.com/products/anti-htlv-type-i-p19-clone-tp-7-100-g
Rabbit, Mouse IgG HRP Linked Whole Ab (Cytiva Cat# NA934-1ML, NA931-1ML); Manufacturer's website: https://www.cytivalifesciences.com/en/us/shop/protein-analysis/blotting-and-detection/blotting-standards-and-reagents/amersham-ecl-hrp-conjugated-antibodies-p-06260
GM130 polyclonal antibody, Alexa Fluor 647 (Thermo Fisher Scientific Cat#PA1-077-A647); Manufacturer's website: https://www.thermofisher.com/antibody/product/GM130-Antibody-Polyclonal/PA1-077-A647
LAMP2 monoclonal antibody, Alexa Fluor 647 (Thermo Fisher Scientific Cat#A15464); Manufacturer's website: https://www.thermofisher.com/antibody/product/LAMP2-Antibody-clone-H4B4-Monoclonal/A15464
Goat anti-Rabbit (H+L) Cross-Adsorbed Secondary Antibody, Alexa Fluor 488 (Thermo Fisher Scientific Cat#A-11008); Manufacturer's website: https://www.thermofisher.com/antibody/product/Goat-anti-Rabbit-IgG-H-L-Cross-Adsorbed-Secondary-Antibody-Polyclonal/A-11008
Goat anti-Mouse (H+L) Cross-Adsorbed Secondary Antibody, Alexa Fluor 594 (Thermo Fisher Scientific Cat#A-11005); Manufacturer's website: https://www.thermofisher.com/antibody/product/Goat-anti-Mouse-IgG-H-L-Cross-Adsorbed-Secondary-Antibody-Polyclonal/A-11005
Alexa Fluor 594 anti-human CD4 (BioLegend Cat#300544); Manufacturer's website: https://www.biolegend.com/en-us/products/alexa-fluor-594-anti-human-cd4-antibody-9433
APC/Fire 750 anti-human CD3 (BioLegend Cat#317352); Manufacturer's website: https://www.biolegend.com/en-us/products/apcfire750-anti-human-cd3-antibody-17500
APC/Fire 750 Mouse IgG2a, k isotype control (BioLegend Cat#400284); Manufacturer's website: https://www.biolegend.com/en-us/products/apc-fire-750-mouse-igg2a-kappa-isotype-ctrl-13010
Brilliant Violet 421 anti-human CD25 (BioLegend Cat#302630); Manufacturer's website: https://www.biolegend.com/en-us/products/brilliant-violet-421-anti-human-cd25-antibody-7139
Brilliant Violet 711 anti-human CD8a (BioLegend Cat#301044); Manufacturer's website: https://www.biolegend.com/en-us/products/brilliant-violet-711-anti-human-cd8a-antibody-7929
Annexin V Alexa Fluor 488 ready flow conjugate (Thermo Fisher Scientific Cat#R37174); Manufacturer's website: https://www.thermofisher.com/order/catalog/product/R37174?SID=srch-hj-R37174

Eukaryotic cell lines

Policy information about cell lines and Sex and Gender in Research

Cell line source(s)	293T (ATCC; CRL-3216)<br>Jurkat (ATCC; TIB-152)<br>HL-60 (ATCC; CCL-240)<br>MT-2 (AIDS Research and Reference Program, NIAID- currently BEI Resources Cat#ARP-237)<br>Jurkat Tax Tet-on were provided by Dr. Warner Greene (PMID: 16105841)<br>HTLV-1 transformed T cell lines (C8166, HUT-102 and SLB-1) were obtained from Dr. Shao-Cong Sun (PMID: 9694868).<br>TL-OM1 and MT-1 cell lines were provided by Dr. Michiyuki Maeda (PMID: 15878527).<br>ATL-2S cells were provided by Dr. Masao Matsuoka (PMID: 14991578).<br>JET cells were provided by Dr. Jun-ichi Fujisawa (PMID: 29186194).
Authentication	Tax+ (MT-2, C8166, HUT-102, SLB-1) and Tax- (TL-OM1, MT-1, AYL-2S) cell lines were authenticated by western blotting for Tax protein. None of the other cell lines were authenticated.
Mycoplasma contamination	Cell lines were routinely tested for mycoplasma contamination using MycoAlert Plus Mycoplasma Detection Kit (Lonza LT07-703). Cell lines were only used for experiments if tested negative for mycoplasma.
Commonly misidentified lines (See ICLAC register)	Not listed in ICLAC.

Plants

Seed stocks	*Report on the source of all seed stocks or other plant material used. If applicable, state the seed stock centre and catalogue number. If plant specimens were collected from the field, describe the collection location, date and sampling procedures.*
Novel plant genotypes	*Describe the methods by which all novel plant genotypes were produced. This includes those generated by transgenic approaches, gene editing, chemical/radiation-based mutagenesis and hybridization. For transgenic lines, describe the transformation method, the number of independent lines analyzed and the generation upon which experiments were performed. For gene-edited lines, describe the editor used, the endogenous sequence targeted for editing, the targeting guide RNA sequence (if applicable) and how the editor was applied.*
Authentication	*Describe any authentication procedures for each seed stock used or novel genotype generated. Describe any experiments used to assess the effect of a mutation and, where applicable, how potential secondary effects (e.g. second site T-DNA insertions, mosiacism, off-target gene editing) were examined.*

Flow Cytometry

Plots

Confirm that:

☒ The axis labels state the marker and fluorochrome used (e.g. CD4-FITC).

☒ The axis scales are clearly visible. Include numbers along axes only for bottom left plot of group (a 'group' is an analysis of identical markers).

☒ All plots are contour plots with outliers or pseudocolor plots.

☒ A numerical value for number of cells or percentage (with statistics) is provided.

Methodology

Sample preparation	Refer to Methods section for details about specific procedures.
Instrument	Cells were analyzed using BDFACS Symphony A3 and FACS Calibur.
Software	BDFACS Diva and BD CellQuest software was used for collecting data. FlowJo 10 was used for analyzing collected data.
Cell population abundance	This study did not involve any cell sorting.
Gating strategy	Gates were set using appropriate controls. Identical gating strategies were applied to all samples. Gating strategies are provided in Supplementary Information.

☒ Tick this box to confirm that a figure exemplifying the gating strategy is provided in the Supplementary Information.
